# Why should i comply? Sellers' accounts for (non-)compliance with legal age limits for alcohol sales

**DOI:** 10.1186/1747-597X-7-5

**Published:** 2012-01-23

**Authors:** Jordy F Gosselt, Joris J Van Hoof, Menno DT De Jong

**Affiliations:** 1 Department of Communication Sciences, University of Twente, Enschede, the Netherlands

**Keywords:** compliance, age limits, alcohol sales, availability, adolescents, vendors

## Abstract

**Background:**

Availability is an important predictor of early and excessive alcohol consumption by adolescents. Many countries have implemented age limits to prevent underage purchases of alcohol. However, shop-floor compliance with these age limits appears to be problematic. This study addresses the issue of non-compliance with age limits. Which measures do vendors take to avoid underage alcohol sales, and what do they report as important reasons to comply or not with age limits for alcohol sales?

**Methods:**

Open-ended telephone interviews were conducted with store managers selling alcohol (N = 106). Prior to the interviews, all outlets were visited by an underage mystery shopper in order to measure compliance with the legal age limits on alcohol sales. The interview results are compared against actual compliance rates.

**Results:**

Several measures have been taken to prevent underage sales, but the compliance level is low. Furthermore, open coding resulted in 19 themes, representing both valid and invalid arguments, that vendors mentioned as relevant to their decisions of whether to comply with the law. Compliance with age limits is dependent on the knowledge of the rules and the ability and motivation to follow the rules. The ability aspect in particular seems to be problematic, but in many cases, the motivation to actively comply with the age limits is lacking.

**Conclusions:**

To enhance compliance, it is important to raise the awareness of the importance of age limits and to connect possible violations of the regulations to negative consequences.

## Background

Reducing adolescents' consumption of risky substances such as alcohol, tobacco and illicit drugs is an important challenge for national and local governments, politicians, policy makers, health professionals and academics. The age at which people start using risky products appears to be predictive of their consumption levels and related problems in later years [1]. A wide range of risk and protective factors affect the onset and escalation of adolescent risk behaviors [2-4]. Availability, in general, is considered an important predictor of adolescents' initial consumption and consumption patterns, along with the consumption-related damage they cause [5-9].

Although delaying the onset of consumption is associated with positive effects, modern societies make these substances widely available. In an attempt to decrease the availability of these substances, many countries have introduced special rules in the form of age limits. Such regulations prohibit the sale of substances to customers below a certain age, which should result in an overall reduction in the commercial availability of these products for adolescents and a prevention of problems among youth who have already started consumption.

### Compliance with age limits

Whether minors succeed in obtaining age-restricted products depends not only on the legislation but also on the extent to which vendors (managers and/or sales employees) comply with these age restrictions in their daily practices within their stores. Compliance studies conducted worldwide [10-17] show that compliance is actually low, although differences in compliance rates between countries exist [16,18,19]. Several studies also identified context factors that may influence compliance with age limits, such as characteristics of the general establishment (e.g., type of license, location of the establishment), the interior of the premises (e.g., busyness, signs in the store), vendor characteristics (e.g., gender, age), buyer characteristics (e.g., gender, educational level), and characteristics associated with the purchase attempt itself (e.g., time of visit) [11]. In general, especially establishment characteristics (e.g., type of business, license type) and characteristics associated with the purchase attempt itself seem to be related to the likelihood of illegal alcohol sales to minors, more so than for example any vendor or buyer characteristics [11]. Alcohol control policies can be effective in preventing alcohol-related harms, considering that it will be easier to influence licensed occupational behavior than it is to influence the behavior of private customers [20,21]. In compliance literature, the effects of several interventions aimed at improving compliance were tested using pre- and post-intervention assessments, for example for educational and training interventions [22-26], raising the purchase age [23], intensifying enforcement [16,22,24], personal feedback on shop-floor compliance [25], mass media campaigns [26], or technical solutions [27,28]. To summarize the main findings: only providing information (training and campaigns) has minimal effects, while raising the minimum legal drinking age, enforcement, feedback and technical solutions appear promising interventions to increase age verification and subsequently compliance.

To date, the compliance literature has mainly focused on whether vendors and consumers comply with age limits and the factors that may influence compliance. To further our insights on compliance with age restrictions, it seems useful to also consider compliance from a more diagnostic perspective. To establish highly effective systems of age limits, it is important to know not only whether there is compliance but also why there is compliance or non-compliance, along with the way in which age limits are viewed by the most prominent actors involved: the vendors working in outlets where risky products are sold. Diagnostic information helps us to uncover the strengths and weaknesses of the system in order to increase compliance.

### Compliance with rules

In the fields of the environment, food safety, occupational health and safety, and financial services, the question of how to influence the acceptance of and compliance with rules is a prominent one [29-32]. In the case of the availability of detrimental substances, factors that may be conditional on an industry level include sufficient overlap of private interests with public interests, the existence of pressures to comply, a small number of actors in a highly organized and homogeneous sector, and the degree of social responsibility within the sector [33]. Poor performance of certain (groups of) actors and of the whole regulatory system can be counterproductive in regulatory practice [33-35].

Whether the industry complies with the rules may be affected by the supportiveness of the outlet management [36] and by the personal support from vendors for these rules. Three conditions are important [37-40]. First, the regulated parties must know and understand the rules. Clear and uniform communication about the rules is therefore essential. Second, it is important that the parties are able to follow the rules. For example, resistance to the rules that vendors may encounter in practice (e.g., a situation in which minors threaten the staff of a store after a refusal) may diminish the ability to follow the rules. Third, the regulated parties have to be willing and motivated to comply. Here, their attitudes toward compliance are important [39,41-44], while the motivation may also be related to the prominence of sanctions and/or enforcement [45].

In the domain of detrimental media (e.g., games or movies with violent content), we quantitatively examined the determinants of vendors' compliance with age limits, resulting in three factors that appeared to be related to vendors' self-reported degree of compliance not to sell detrimental media to customers who are too young according to an age classification [46]. The first factor is their personal acceptance of the systems. The individual perceptions of vendors, more than the perceptions on the level of the store, may affect their willingness to comply with the rules. A second important factor involves the perceived legal basis for the age restrictions. The willingness of vendors to comply increases when they are aware that there is a legal basis for the system. The third factor is the perceived degree of (external) surveillance. Compliance increases when vendors are aware of the possibility of internal and external monitoring activities. It is conceivable that the factors that were important for detrimental media do not reflect the issues that are relevant in other contexts (for example, due to different regulations or different detrimental consequences after consumption). In the current study, we explore compliance-related issues in the context of alcohol sales.

### The Dutch situation on alcohol sales and age limits

As in many other countries, in the Netherlands, age limits have been implemented for several risky products such as alcohol, tobacco, detrimental media, gambling products and marijuana. For alcohol, two age limits are used: 16 years of age for soft alcoholic beverages (< 15% alcohol, and some distilled wines, such as port and sherry), and 18 years of age for strong alcoholic beverages (> 15% alcohol). Sales personnel in supermarkets, liquor stores, and the catering industry are obliged to ask for someone's identification if there "could be any doubt about the age of the potential customer." It is also forbidden to sell alcohol to someone who is older than 16 years (or in the case of strong alcohol, older than 18 years) when the liquor is apparently intended for a person who may be under 16 or 18 years of age (this is also known as secondary purchasing). In those cases, the age of the second person must also be verified. Because sales personnel are the single party responsible for compliance, they are crucial for a proper functioning of the age limit regulations. In a recent study, 100% of the interviewed sales personnel in supermarkets, 99% of the personnel in liquor stores and 93% of the vendors in bars claim to comply with the age limit for alcohol sales [47]. Decoy compliance studies, conducted during a four-year period and consisting of hundreds of underage mystery shopping alcohol purchase attempts, however, showed an average Dutch compliance level of 15% [26].

The Dutch compliance level on alcohol sales is in line with USA and UK findings. Studies conducted in the United States show compliance levels varying from 3% up to 74% [11,22,48-51] and in the United Kingdom a 17% compliance was found [52]. Within the Netherlands, the Dutch Food and Consumer Product Safety Authority enrols the enforcement on (among others) compliance with the legal age limits on alcohol sales. Without prior notice the FCPSA visits outlets selling alcohol containing products and observes whether under aged people buy alcohol. If the authority observes a violation of the law it may warn or fine the vendor and/or store owner (in the current legislation only vendors are liable to the age limits). Structural offence with this legislation may result in an official procedure aimed at expropriation of the alcohol sales licence.

In this study, we qualitatively explore the factors that may affect compliance with the age limits in the context of the Dutch alcohol legislation. Interviews were conducted with managers of different types of alcohol outlets to gain diagnostic insight into compliance-related issues in the daily practice of the shop floor.

## Methods

To learn what alcohol vendors experience in their daily practice and how they speak about age limits from the perspectives of their own values and experiences, we interviewed 106 store managers on compliance-related issues. The interviews reported here were part of a larger study [25] that took place within one region in the Netherlands, consisting of nine municipalities that contained approximately 350 alcohol outlets in total.

In that study, we first conducted underage purchase attempts to determine compliance with the 16-year age restriction for alcohol sales. Taking into account the influences of opening hours (e.g., no night bars) and seasons (e.g., the specific opening times for beach pavilions and sports clubs), as well as the likelihood that an outlet may be visited by a 15-year-old adolescent in daily practice (which excluded most bars), 146 alcohol outlets --37 supermarkets, 26 liquor stores, 46 cafeterias (privately owned fast-food restaurants) and 37 bars--were at randomly selected and subsequently visited by four underage adolescents (two boys and two girls, all 15-year-olds who looked 'average') who tried to buy soft alcoholic beverages, following two types of scripts, based on the Dutch standard, as being used more than 7,000 times so far [17]. These scripts were trained before the visits took place. Within the supermarkets, liquor stores and cafeterias the 15-year-old adolescent entered alone and chose one alcoholic beverage--beer for male and premix drinks for female mystery shoppers (both containing about 5% alcohol), and took it to the checkout. If they were asked whether the alcohol was for personal use, the mystery shoppers would answer affirmatively. If they were asked about their age, they would lie and claim that they were 16 years old. If they were asked for an ID, they would show their real ID. Within the bars two mystery shoppers entered the location and ordered two beers. The rest of the script was the same as the other scripts. The adolescents were recruited by their own high-school teachers, and informed parental consent was obtained. The protocol was approved by the Ethical Commission of the Faculty of Behavioral Sciences of the University of Twente. Then, in interviews, we examined the effects of an intervention that consisted of feedback on the store's own compliance, and questions were asked concerning compliance-related issues in general. The current article focuses on the latter part of the interview.

### The interviews

After the under-aged purchase attempts, all alcohol outlets visited were contacted by telephone and asked to participate in a telephone interview. After a short explanation of the research, 106 of the 146 alcohol outlets agreed to an interview. The 40 outlets that were not interviewed included cafeterias of which the owner or vendor did not speak Dutch and supermarket chains that only had a central company helpdesk. None of the alcohol outlets approached (n = 106) refused participation for the interview, so there was no selection bias. Because of the delicate nature of the topic and some of the questions, we assured absolute confidentiality and anonymity. Based on a semi structured checklist all answers were written down.

### Procedure

The interviewers (all research assistants of the University of Twente) first made sure that they were speaking with the store owner or manager. The interview began with a short introduction of the mystery-shopping research and the outcome of the compliance check within the interviewee's own store. The interview scheme was open ended, which allowed interviewees to set forth their own views and experiences. Three general questions on compliance were asked: (1) Do you, or did you, undertake any action to prevent the sale of alcohol to minors? (2) What are the main reasons to comply or not with the law that prohibits the sale of alcohol to minors? and (3) What would help to improve compliance?

### Data analysis

Following the cross-sectional code and retrieve principle [53], a qualitative analysis of the interviews by two coders who were extensively trained was organized to uncover the main topics and categories mentioned by the interviewees across the entire data set. After becoming familiar with the material, the coders identified recurring themes and ideas, which led to a general framework. Then, all variables were carefully defined in a codebook that could be referenced at any time. Substantial coding practice took place to ensure that coders understood what qualified as evidence of each variable. Twenty-eight percent of the total sample of interviews was analyzed by two coders and satisfactory intercoder reliability was achieved. After that the coding of the remaining interviews took place by one coder based on the codebook. This open coding, in which all speech was coded, generated 1,492 codes spread over 33 categories (some of them with different subcategories). Transcripts were uploaded into Atlas.ti, creating an overview of how the interviewees thematically structure their thoughts about and experiences with the subjects under study.

### Coding

For all variables, the Cohen's kappa was found to be acceptably high. The first nine codes cover the measures that are taken by the interviewees to prevent underage sales. The initial kappa for the measures-codes was .76. After extensive discussion and several adjustments in the codebook, the (unweighted) kappa increased to .93. Then, 19 categories cover the compliance related issues that were put forward by the interviewees. Further, *don't know *answers and answers that state that compliance is *no issue *within the store were coded as well. These 21 categories on the compliance-related issues reached a Cohen's kappa of .85. We multicoded all utterances in these 21 categories with one (or more) of the following three main categories that we identified, under which all answers on compliance could be classified: (1) reason(s) for compliance, (2) reason(s) for non-compliance, and (3) solution(s). These three main categories reached full agreement (Cohen's kappa = 1.0).

## Results

Below, we will first discuss the results of the underage purchase attempts, followed by the measures alcohol outlet managers report taking in order to avoid alcohol sales to minors. Second, we will focus on the reasons they give for compliance or non-compliance, along with the solutions they propose.

### Actual compliance with alcohol legislation

The overall compliance (N = 146) was 18.5%, indicating a low compliance rate within this region, although it is in line with other compliance studies conducted in the Netherlands. Compliance was highest in liquor stores (54%), followed by supermarkets (19%), bars (16%) and cafeterias (0%). In the majority (96 times) of all visits, no intervention (asking for age and/or ID) took place. Of the 119 stores in the non-compliance condition, we interviewed 82 (69%) and of the 27 stores that did comply we were able to interview 24 (89%). Main reason for drop out in the interviews were supermarkets which were not reachable by phone and restaurant owners not speaking Dutch (mainly Chinese ones, generally also not complying). The compliance rate within the stores that were interviewed (N = 106) was 22.6%.

### Measures to increase compliance with age limits

Eight categories of measures were reported to increase compliance with age limits, resulting in 123 measures in total. There were only a few outlets (n = 18) that reported not taking any type of measure to assure compliance with the rules. Vendors working in cafeterias, in particular, indicated that no actions were needed, as they would encounter young customers in their establishment only occasionally (or not at all), because no alcohol is sold (which is not the case, as we did buy alcohol before), or because vendors purport to know (the age of) their clientele. In general and within all outlet types, most vendors focus on asking for the age of an (under age) buyer or for some type of identification. Identification either is needed for all customers (occasionally, because the personnel are required to do so), or only when a customer looks younger than a certain age (whereby varying age limits are enacted, ranging from 16 to 40 years of age). Some interviewees said that age-verification activities occur only from time to time and not on a consistent basis. Then, in most cases, not being able to show an ID means that no transaction will take place, according to these vendors. Despite all of the measures that vendors purported to take, the underage purchase attempts show that compliance is low nonetheless. In 96 of the 106 visits, the age of the young customers was not verified at all. Table 1 gives an overview of all of the measures mentioned by vendor, according to outlet type, that should lead to age verification.

**Table 1:** Current measures taken to prevent underage sales in supermarkets (Sup), liquor stores (Liq), cafeterias (Caf) and bars (Bar).

Measure	Outlet type				Total
	
	Sup	Liq	Caf	Bar	
# interviews	29	24	32	21	106
Training own personnel	25	20	6	3	54
Information to the public	3	8	6	2	19
Setting higher age limit(s)	0	8	2	4	14
Internal support systems	4	4	0	3	11
Limiting secondary purchasing	2	3	0	2	7
Bouncers	0	0	0	5	5
Paying attention to other cues	0	1	2	0	3
Other actions	2	2	2	4	10
Total	36	46	18	23	123
Nothing (no need)	1	1	11	5	18

*One outlet may have taken more than one measure*.

To encourage vendors to verify customers' ages, the outlet personnel receive training (n = 54). Especially within supermarkets and liquor stores, cashiers receive training on the rules/legislation on age restrictions. This training is performed primarily when a new employee starts; however, within some stores, this training has a more structural nature (for example, by means of monthly/weekly updates or special 'cash-register evenings'). Different forms of training exist: for example, in some stores, the vendors receive training from a colleague, while in other stores, training occurs by means of instructional DVDs, instructional letters, reminders, or aid systems on this subject. In supermarkets, some vendors said that the personnel could earn a certificate after an (online) course on age limits. For the most part, training or information sessions are initiated by the head office. Further, personnel is sometimes made aware of compliance issues by (camera) surveillance or by underage purchase attempts commissioned by the management.

In addition to training the personnel, managers use several other measures to enable, force and remind the personnel to execute their tasks and obligations, for example, by providing information that is aimed at the public (n = 19). Liquor stores and cafeterias often use signs in the store, posters, stickers, websites, self-made notes, and/or letters informing the (young) public about the alcohol legislation and the fact that identification must be shown. This information is placed near the cash register or at the entrance of the store. Further, some of the stores employ a higher age limit standard than that prescribed by law (n = 14). Signs then communicate that anybody appearing below the age of 20 (in supermarkets) or 25 (in liquor stores) must show ID. In bars, higher age limits are sometimes noted at the entrance. Additional support systems are sometimes used (n = 11), including a conversion table to help the cashier calculate the customer's age based on his/her date of birth, a cash-register system that beeps whenever an age-restricted item is scanned (e.g., alcohol, tobacco, gambling products), colored arm bands or stamps in bars that note the buyer's age, and a document that depicts images of all legal forms of identification. In one sports bar, all members' names are provided on a digital membership list noting the customer's age. To decrease secondary purchasing, in some stores (n = 7) the personnel is instructed to detect secondary purchasing (for example, refusing a sale to a group of young people when only one is old enough legally) and to decline sales in cases where a young customer claims that the alcohol is for his/her parent(s). Five times the presence of bouncers (personnel stationed at the front door to verify the age of customers before they enter the premises) was mentioned, and three times other cues are noted that might indicate that the buyer is underage (such as whether he/she was riding a scooter, which are typically used by persons under 18 years of age). Other measures implemented (n = 10) include having the sales personnel sign a contract stating that they are familiar with all rules concerning age restrictions, conducting breathalyzer tests on underage customers, and, in bars, making soft drinks cheaper than alcoholic beverages.

The stores that did comply in the mystery shop study are responsible for 39 (= 32%) out of the 123 measures in total that were reported to be taken in order to prevent underage sales. Within this group especially 'setting higher age limits' and 'bouncers' were predominantly mentioned as measures being taken, while also 'internal support systems', 'information to the public' and 'limiting secondary purchasing' were mentioned regularly. However, these figures should be interpreted with care because of the small number of cases.

### Compliance with the rules and solutions to increase compliance

In the second part of the interview, the reasons for compliance and non-compliance were addressed, along with the vendors' proposed solutions. Nineteen categories were identified of compliance-related issues mentioned by the interviewees. Table 2 gives an overview of the frequency of utterances for each coding, classified under *Knowledge*, *Ability*, *Motivation *or *Other*. Apart from six interviewees who indicated that compliance with age legislation is not a problem for their businesses, the number of quotations presented in Table 2 gives reason to believe that age limits are an important topic for vendors of alcohol.

**Table 2:** Reasons for compliance (Com) and non-compliance (NonC), and solutions (Sol).

		Answer		
		Com	NonC	Sol
Knowledge				
	(Knowledge of) rules	1	7	14

Ability				
	Secondary purchasing	0	39	2
	Estimating buyer's age	0	22	9
	Fear to intervene	0	20	5
	Aggression	0	23	1
	Reluctance to ask	0	9	6
	Relationship to buyer	0	10	2
	Busyness/time	0	12	0
	Unwillingness to ask again	0	9	1
	Use of fake ID's	0	5	3
	*Total *	*0*	*149*	*29*

Motivation				
	Intrinsic support	71	9	13
	Responsibility	8	38	33
	Blaming others	0	22	8
	Law-abiding nature	22	0	2
	Financial reasons	17	2	1
	Reputation	3	0	0
	Surveillance	0	5	4
	Integrated approach	0	0	4
	*Total *	*121*	*76*	*65*

Other		3	21	24

Total		125	253	132

No issue		0	6	0
Don't know/no answer*		6	13	17

*No answer was given on the question regarding why one would comply or not.

First, we will describe the reasons that vendors give for complying with the rules not to sell alcohol to persons who, according to law, are underage. Then, we discuss the arguments vendors give for non-compliance, and finally, the solutions that were proposed are discussed.

#### Why comply: reported reasons for complying with age limits (n = 125)

The most prominent reason to comply with the alcohol legislation relates to motivational arguments (n = 121), such as intrinsic support for this specific law (n = 71). Support is a result of concerns about the physical and mental health (i.e., brain damage) of minors, alcohol abuse (e.g., intoxication, experimentation with other substances), and nuisance (e.g., vandalism, public order). There is also concern about the current consumption pattern (*"Nowadays, young people already drink too much and too often" *and *"Nowadays, they start at a much younger age"*). Others state that they want to prevent young people from drinking too much; however, *"[...] a beverage now and then is OK." *Some did not specify why they support this law, instead arguing *"[...] not to sell because the law dictates us to do so." *This reason overlaps with the law-abiding reason, which was also frequently mentioned (n = 22): *"You are supposed to comply with the law" *and *"Rules are rules." *Many vendors refer to their own children and, when doing so, relate consumption to availability: *"I have children myself, and I don't want them to start at an early age." *The most mentioned financial reason (n = 17) to comply is, not surprisingly, to avoid fines. Responsibility arguments (n = 8) represent a feeling of social responsibility and a role-model function: *"Apparently, parents and the kids no longer are able to control themselves [...]*." Only three vendors mention the reputation argument: *"[...] it can give us a bad name." *Surveillance was not mentioned at all. Thus, in general, vendors seem motivated to prevent or discourage (first) use and for that reason, do not sell to underage customers.

Concerning the knowledge of the rules (n = 1), only one employee said that the personnel is well informed about compliance issues. Other reasons (n = 3) that were mentioned relate to the public nuisance associated with alcohol consumption. The vendors that complied in the mystery shop study predominantly indicated that compliance is a result of their intrinsic support and because they feel responsible.

#### Why sell: reasons for non-compliance (n = 253)

Aside from the thirteen interviewees who reported no reasons for non-compliance, most answers related to ability (n = 149), as can be seen in Table 2. Secondary purchasing (n = 39) is the most prevalent problem reported, which influences ability. Non-compliance results because older friends are deployed (*"[...] It is difficult when, after refusal, a young customer sends in a friend that buys him/her the alcohol. In those cases, we cannot do anything but sell the alcohol"*), or even parents (*"What complicates compliance is when [...] parents buy alcohol for their child"*). This argument is not valid (meaning not in line with the legal rules and regulations of the Dutch alcohol law) because, according to the law, in these cases, the age of both customers should be verified. Thus, next to ability, knowledge is not optimal either. A second problem affecting ability that vendors mentioned includes the aggression argument (n = 23). Young clients that become aggressive, intimidating, annoying or violent may lead to non-compliance: *"[...] it is kind of intimidating to ask for one's age. This regularly leads to aggressive youngsters and problems. [...] Of course, it is inexcusable that this affects compliance, but it does make it difficult." *Aggression mainly occurs after refusal of sale or when the customer is old enough but must show his/her identification nevertheless. Third, estimating the age of the customer (n = 22) is considered to be difficult. The young age of the sales personnel is often explicitly regarded as an additional complicating factor. Estimating the age is regarded difficult because children nowadays look older (especially girls), some use makeup to look older, they may lie about their age, and they are allowed to go out sooner. These vendors did not mention proper age verification as the only solution. Also, being afraid of intervening (n = 20) may result in non-compliance. In all of these cases, the young age of cashiers plays an important role (*"What complicates compliance is that cashiers are very young, and when they have to ask customers, who are the same age, for ID, they find this threatening"*). Another issue that was raised is busyness within a store (n = 12), especially in places where most of the clientele is of a young age. Sixth, age verification and/or refusal of sale is said to be difficult when the vendor and buyer know one another personally. Especially when personnel are young, there is a chance that they will encounter underage friends at the register (n = 10). For older personnel this may also play a role because *"you don't want to ask people you already personally know for identification. That is unpleasant and therefore something we don't do." *Following this line of reasoning, interviewees stated that it is uncomfortable to repeatedly ask for identification (n = 9): *"You don't want to ask people you know for identification over and over again; that is really annoying." *Furthermore, intervening in general is considered to be quite unpleasant (n = 9) as *"you might insult someone who is old enough" *and *"it is unpleasant that, every time, we have to act like a police officer." *Five times the use of false identification cards was mentioned as a factor that makes age verification problematic. Underage customers' use of false identification cards may result in illegal sales without the vendor's knowledge.

After the ability aspects, the motivational dimension (n = 76) seems to be important in the decisions of vendors not to comply with the rules. Vendors (n = 38) do not think that they are (solely) responsible when it comes to underage alcohol consumption. The role of the parents was mentioned explicitly: *"I think also the parents are responsible. Outlets may make all the efforts they can; parents still are responsible for their child." *Some vendors mentioned the parents' irresponsible behavior (parents that buy the liquor for their own child), while others posited that the adolescent is also responsible, both in terms of their own health as well as in a legal respect: *"An important bottleneck is that the one that buys the alcohol is not punishable." *In addition to the parents and minors themselves, vendors also found that other vendors are responsible for violations of the rules (n = 22; i.e., blaming others): *"If they cannot buy it here, they will succeed elsewhere." *Neighborhood supermarkets and bars in particular were thought not to act in accordance with the law because of *"their young personnel" *and *"their low prices." *Also, non-commercial private drinking places (so called "barracks") and young friends are to blame (see "secondary purchasing"). Vendors questioned the necessity of rules that should protect minors (n = 9): *"I wonder how bad alcohol actually is for minors, as others also buy them the alcohol,", "We shouldn't stimulate underage alcohol consumption, but on the other hand, we also should not forbid it. [...] It's better to let them get used to alcohol than when they go wild later," *or *"I find it disturbing that as a bar, you are judged on when you accidentally sell to a minor once. Even when you show that you are very busy with this whole legislation, it doesn't matter." *Five reasons vendors gave for not complying with age limits are related to the lack of internal and external control, and two vendors mentioned financial reasons, given that they lose profits when they refuse a sale.

Furthermore, compliance was said to be problematic because of knowledge of the rules (n = 7). There are two age limits, and sales personnel are obligated to verify someone's age (by identification card) if there "could be any doubt about the age of the potential customer." The rules are considered *"vague." *Additionally, the vendors expressed confusion about the existence of strong liquors that contain less than 15% alcohol and, therefore, legally should be regarded as soft alcoholic beverages. Furthermore, temporary employees were said not to possess sufficient knowledge of the rules. Interviewees also stated that the information supplied by managers should be improved.

Other reasons that were mentioned (n = 21) involve adolescents going out at a much younger age than they used to; adolescents who think that "*they are allowed to buy everything, not knowing that we get in trouble*"; that despite all of the vendors' efforts, it will always be possible for a youngster to obtain an alcoholic beverage; and, especially, that the personnel are not sufficiently attentive: "*A bottleneck is that the employees are the ones who have to actually do it*."

In sum, the arguments mentioned here suggest that not all vendors wholeheartedly support the age limits. Furthermore, many of the arguments used here represent shifting responsibility, blaming others and defending their own low compliance rates.

#### Solutions (n = 132)

After asking vendors to elaborate upon the problems they encounter, we asked them what they see as possible solutions for a higher compliance rate. There were interviewees who did not suggest any type of solution that could enhance compliance (n = 17). Of the vendors that did offer solutions, most answers focused on motivational aspects (n = 65), shifting responsibility to parents and/or adolescents and calling for an awareness campaign to increase compliance (n = 33). Thirteen responses related to the intrinsic support for an age limit system, especially for the age limits that are currently being used. Vendors propose to introduce one age limit that will apply to both weak and strong alcoholic beverages: *"That distinction is really ridiculous. Twenty beers will also get you drunk, even more so than three Bacardi cokes." *The interviewees also emphasized the importance of surveillance and that the possibility of fines should be communicated (surveillance: n = 4), especially to other stores (blaming: n = 8). Customers should be made to show ID, and vendors and the public should become accustomed to the idea that everyone (below a certain age) must always show an ID when buying a risky product. Four times it was stated that more collaboration is needed between all of the relevant actors, such as police, the municipality, vendors, schools, and parents (i.e., an integrated approach). Related to law-abiding, two interviewees stated that the only solution is simply to obey the law.

Regarding solutions concerning ability (n = 29), some interviewees suggested permitting a maximum number of adolescents in the store to avoid secondary purchasing. They also recommended the issue of special alcohol cards to ease age verification and further training of cashiers to diminish their fear to intervene.

Interviewees also suggested providing more information and education about the risks of early alcohol consumption to increase knowledge (knowledge of rules: n = 14). This information should be aimed at adolescents and/or parents in the form of school programs, national campaigns or in-store education.

Other solutions (n = 24) that were mentioned mainly involve (continuing) the verification of age and/or ID (*"You just keep asking"*), limiting the temptations of alcohol (advertising bans), and eliminating alcopops.

In sum, the solutions mentioned seem to suggest a shifting of responsibility to parents, adolescents, or other vendors. Only a few vendors suggested solutions that directly affect their own daily practice, such as measures of intensified surveillance.

## Discussion

The prevention of alcohol-related health problems among youngsters has become of increasing concern in many countries. To reduce underage consumption, the literature shows that it is important to decrease availability. Therefore, worldwide, many governments have implemented age limits to prevent underage sales of substances that are considered to be detrimental for the physical or mental health of minors. Literature also shows that such limits, set out in regulations (which often differ between countries and even between products) are effective only when compliance with these rules is sufficient.

In this study, managers and vendors of supermarkets, liquor stores, cafeterias and bars listed a broad range of measures they purport to take within their store in order to comply with the legal age limits on alcohol sales. Within the 106 stores, 54 stores have trained personnel, 19 stores give information to the public, 14 stores employ a higher age limit standard than that prescribed bylaw, 11 stores use additional internal support systems, 7 stores actively try to limit secondary purchasing, 5 bars employ bouncers, 3 stores pay attention to other cues that may reveal that the customer is too young, and 10 stores use other actions. Moreover, the more measures a store takes seems to positively influence compliance, although more research is needed on the relationship between the amount of actions undertaken and the level of compliance. Only 18 of the 106 interviewed store managers and vendors (mainly from cafeterias) do not take any measures to improve compliance with age limits. So, vendors claim to use several measures to secure age verification of young customers. Despite all of the claimed measures, however, compliance is only 18.5% on average, which demonstrates that the measures are not sufficient to prevent illegal alcohol sales to underaged customers. Furthermore, some of the actions seem to be simple excuses and might be categorized as 'window dressing'. Informing the public about the rules may increase awareness among the public. However, this may also indicate shifting responsibility away from the verification obligation that vendors have. In all, training personnel, employing higher age limits, using internal support systems, hiring bouncers and taking other actions apparently do not lead to sufficient compliance levels.

The open interviews with the vendors resulted in nineteen categories that cover several issues related to (non-)compliance with age limits for alcohol sales. In the introduction, we identified three general determinants of compliance. According to compliance literature [37-40], all people involved should first know and understand the rules. Vendors said little regarding self-reported knowledge of the rules. However, during most interviews, the age limits that are active were mentioned, and many vendors who we interviewed also explicitly mentioned the age-verification activities and the harmful effects of underage alcohol consumption. In some cases, however, vendors are not properly informed about the rules in terms of, for example, secondary purchasing. Some vendors say that because some rules are 'vague,' compliance is difficult. They suggest that there should be one age limit in the future, instead of the two current age limits on alcohol sales. Without sufficient knowledge of the rules, it is impossible to act according to the rules.

Second, vendors must be capable of following the rules. Vendors mostly mentioned practical barriers that make compliance difficult. These include customers who become aggressive when they are asked to show ID or who are refused alcohol, personnel who are afraid to intervene, and the buyers who use fake IDs. Many of the practical reasons given, however, can be regarded as invalid arguments, as they show that vendors use excuses to exempt themselves from the obligation to refuse underage sales. The secondary-purchasing issue, which is mentioned many times, is not a valid compliance argument for two reasons: currently, there is little need for secondary purchasing because compliance is low, and contrary to what some vendors seem to think, they are not allowed to sell alcohol to someone who is legally old enough when the alcohol is meant for someone who is too young to purchase according to the law. Problems with estimating the age of a customer can be resolved by simply asking for identification. Busyness within a store, a buyer who is an acquaintance, and unwillingness to ask for identification (multiple times) are not valid arguments and suggest a lack of motivation.

Motivation is the third determinant of compliance: vendors must be willing to comply with the rules [39,41-44]. Based on the interview results, the motivation to comply currently seems to be rather low. The most prominent reason to comply with the rules is based on intrinsic support for the rules and a vendor's law-abiding nature. Intrinsic support results from concerns about the harmful effects associated with early alcohol consumption. Some vendors, however, question the need for rules that decrease the availability of alcohol to minors. Next to intrinsic support, a law-abiding nature ("rules are rules") plays a role among the considerations to comply. However, vendors' sense of responsibility can be considered low. Vendors do not believe that they are solely responsible for the purchases of young customers. This belief leads them to blame others, including parents, the young buyer or his/her friends. Other alcohol outlets are also to be blamed, as they may not comply with the law. Avoiding fines was occasionally mentioned as a reason to comply with the law, while several vendors mentioned a loss of income as a reason for non-compliance. Surprisingly, reputational arguments were rarely mentioned. Working together with other parties (i.e., integrated approach) does not appear to play a prominent role in the motivations from vendors to comply. Moreover, there were very few responses regarding the surveillance aspects, indicating that vendors do not perceive any real chance of being caught for non-compliance.

### Limitations of the study

The data reported here were derived from qualitative interviews, and thus, certain methodological decisions, as well as their influence on reliability and validity, are relevant to address. Regarding representational generalization, we interviewed vendors working within four different types of outlets that sell alcohol: supermarkets, liquor stores, cafeterias, and bars. Although these categories cover most of the selling points available within the Netherlands, due to practical considerations, we did not include some other outlets within the sample, such as sporting canteens. However, the four types of outlets that were included represent the places that are most frequently visited by minors to obtain alcohol. Of course, minors can (and probably will) obtain alcohol within their social environment, such as through parents or friends. In this study, we focused on the commercial availability of alcohol to minors and so only the official channels are relevant.

Second, once we were able to interview vendors, we did not ask for any type of demographic information that could possibly identify the vendor concerned. In our view, keeping the interviewees rather anonymous would decrease non-response and ensure more reliable answers and could decrease socially desirable responses. However, it would be interesting to learn more about the answers given in relation to, for example, the age, gender, and employment position of the interviewee. Further, the fact that the purchase attempt feedback was provided to the vendors just before the actual interview may have influenced the interview responses. For example, it is reasonable to assume that vendors of non-compliant stores may have been defensive. Regarding inferential generalization, as mentioned earlier, next to alcohol, there are many different products provided with age limits. Many differences exist regarding the specific regulations for each of these substances, and subsequently, many differences exist regarding the operationalization per country (e.g., different age limits, different levels of surveillance, degree and height of sanctions). Therefore, although some aspects of the effectiveness of regulations are general--knowledge of the rules as a fundament for compliance, practical issues in the store that may affect actual compliance, and the importance of vendors' personal motivation to comply--the conclusions drawn here do not necessarily apply to other risky substances or to other countries.

### Policy implications

Although it is generally accepted that legal age limits are meant to protect adolescents from health risks, it seems difficult to convince the various actors of their necessity. Governments and healthcare organizations are concerned about the health risks associated with the (early) consumption of alcohol, but industries are primarily driven by profit, and customers may prefer easy access and affordable prices. In the case of age limits, support from the industry is of vital importance. In the best-case scenario, they will follow the guidelines and rules with minimal use of energy and resources. Voluntary compliance is preferred. Involvement with the topic and motivations are also important. Generally, two approaches are possible in changing the behavior of vendors of risky products: confronting them with the positive and negative consequences of their *current *compliance behavior, or supplying them with information on the negative consequences of non-compliance and/or positive consequences of compliance in general.

Compliance with rules depends on knowledge of the rules and the ability and motivation to follow the rules. The ability aspect seems to be especially problematic. However, many of the reasons that were given can be regarded as invalid arguments, as they show that vendors merely offer excuses exempting themselves from the obligation to refuse underage sales. Vendors purport to employ several measures to ease age verification, but the actual compliance figures show that this is not enough. Further, the motivation to actively comply with the age limits seems to be lacking, as vendors feel that parents, children or even other vendors are more responsible or to blame. Surprisingly, reputational considerations do not seem to play a role here. This lack of motivation results in an indifferent attitude with respect to the age limits. Next to increasing knowledge and ability, it is therefore important to raise the awareness of the importance of the regulations and make all parties involved aware of their legal and/or contractual status. This strategy is aimed at reducing ambiguities and increasing people's motivation to comply. However, an exclusive focus on informing and educating retailers (and the public) using methods such as campaign materials or training programs for personnel are not enough to ensure compliance with rules [22,26].

The surveillance aspect was rarely mentioned, indicating that vendors do not receive any feedback regarding their compliance. The instrument 'Table of Eleven,' developed by the Dutch Ministry of Justice, analyzes compliance using eleven dimensions that explain the degree of compliance with laws and legislation [54]. Two groups of dimensions are distinguished: spontaneous compliance dimensions and maintaining dimensions. *Spontaneous compliance *depends on knowledge of the rules, costs and benefits, degree of acceptance, a law-abiding nature of the target group, and non-governmental control activities. These categories indeed show overlap with the utterances that we encountered in our interviews. Very little was said, however, concerning the compliance-maintenance dimension. *Maintaining compliance *is defined as all activities that promote the compliance with laws and legislation, and it mainly depends on the detection and punishment of offences (e.g., sanctions). It is important to connect possible violations of the regulations to negative consequences. Therefore, it appears to be important to develop a valid and visible system of external surveillance, which not only affects the vendors' perceived risk of being caught for non-compliance but also underlines the legal basis and the importance of complying with the age limits. Enforcement and feedback are needed to increase age verification and subsequently compliance [16,22,24,25].

Age limits are promising interventions to decrease underage sales and, consequently, decrease the negative effects that come with underage alcohol consumption. Without compliance with age limits, however, the effectiveness of age limits is limited.

## Competing interests

The authors declare that they have no competing interests.

## Authors' contributions

JG, JvH, and MdJ were responsible for the research design. JG and JvH were responsible for the data collection. JG was responsible for the data-analysis and drafting the manuscript. All authors read, commended on, and approved the final manuscript.

## Authors' information

JG is Assistant Professor at the Department of Communication Studies at the University of Twente (Enschede, the Netherlands). The central topic of his research concerns adolescents and (the availability of) risky products. Several factors are being considered with the use of different research methods. Moreover, studies into the validity and reliability of methods of communication research are of interest.

JvH is Assistant Professor at the Department of Communication Studies at the University of Twente (Enschede, the Netherlands). His research interests include communication science in general, development of research techniques, adolescent (risk) behaviour, impact of both processes of communication and communication message design on audiences in professional and private contexts.

MdJ is a professor of Communication Studies at the University of Twente (Enschede, The Netherlands). His main research interests are methodology of applied communication research, corporate and organizational communication, and the problem of risky products and adolescents.
